# Renal Allograft Rejection: Noninvasive Ultrasound- and MRI-Based Diagnostics

**DOI:** 10.1155/2019/3568067

**Published:** 2019-04-10

**Authors:** Ulrich Jehn, Katharina Schuette-Nuetgen, Dominik Kentrup, Verena Hoerr, Stefan Reuter

**Affiliations:** ^1^Department of Medicine, Division of General Internal Medicine, Nephrology and Rheumatology, University Hospital of Muenster, 48149 Münster, Germany; ^2^Department of Medicine, Division of Nephrology, The University of Alabama at Birmingham (UAB), 35294 Birmingham, Alabama, USA; ^3^Department of Clinical Radiology, University Hospital of Muenster, 48149 Münster, Germany; ^4^Institute of Medical Microbiology, Jena University Hospital, Am Klinikum 1, 07747 Jena, Germany

## Abstract

To date, allogeneic kidney transplantation remains the best available therapeutic option for patients with end-stage renal disease regarding overall survival and quality of life. Despite the advancements in immunosuppressive drugs and protocols, episodes of acute allograft rejection, a sterile inflammatory process, continue to endanger allograft survival. Since effective treatment for acute rejection episodes is available, instant diagnosis of this potentially reversible graft injury is imperative. Although histological examination by invasive core needle biopsy of the graft remains the gold standard for the diagnosis of ongoing rejection, it is always associated with the risk of causing substantial graft injury as a result of the biopsy procedure itself. At the same time, biopsies are not immediately feasible for a considerable number of patients taking anticoagulants due to the high risk of complications such as bleeding and uneven distribution of pathological changes within the graft. This can result in the wrong diagnosis due to the small size of the tissue sample taken. Therefore, there is a need for a tool that overcomes these problems by being noninvasive and capable of assessing the whole organ at the same time for specific and fast detection of acute allograft rejection. In this article, we review current state-of-the-art approaches for noninvasive diagnostics of acute renal transplant inflammation, i.e., rejection. We especially focus on nonradiation-based methods using magnetic resonance imaging (MRI) and ultrasound.

## 1. Introduction

Kidney transplantation (KTx) is the most preferred treatment for patients suffering from end-stage renal disease (ESRD) [1], as it offers enormously better survival rates compared to other renal replacement therapies [2]. Ongoing progress in transplantation medicine results in better success rates of organ transplantation and a prolongation of renal allografts' half-life. [3, 4] Despite the considerable progress of immunosuppressive regimens, acute rejection (AR) still remains a serious issue after KTx, which decreases patient and graft survival rates after its occurrence [5, 6].

AR depicts a condition of sterile inflammation, either antibody or/and T cell mediated.

While the T cell or cellular-mediated rejection (TCMR) usually occurs early after transplantation and is more prevalent, humoral or antibody-mediated AR (AMR) occurs later in fewer patients [7, 8]. Overall, 13–53% of kidney recipients develop an episode of AR within one year after transplantation [9]. The risk of progression to chronic allograft damage with consecutive reduced long-term survival increases with every episode of AR [10, 11]. Chronic allograft failure constitutes still the main cause for death-censored graft loss after KTx [12, 13]. This underlines the importance of early detection and specific treatment for AR.

Transplantation from genetically different tissues involuntarily induces a recipients' immune response against the alloantigens. The formation of antibodies against donor-specific HLA-antigens is a key feature of AMR. Moreover, numerous non-HLA antibodies directed against both alloantigens and autoantigens have been identified to potentially initiate allograft rejection [14]. A critical immunological event inevitably caused by transplantation is related to the ischemia-reperfusion injury of the graft, leading, for example, to an upregulation of expressed HLA antigens in the graft on one side and initiating an inflammatory state on the other side [8]. Moreover, the donor organ transfers immune competent cells to the recipient which may trigger rejection [15].

TCMR is induced by recipient's antigen-presenting cells, which present donor antigens to T-lymphocytes with following activation and differentiation of these cytotoxic T-lymphocytes (CTLs) [16]. Besides CTLs and other leukocyte subtypes, the complement system contributes to the inflammation as well. Once activated, CTLs expanse and differentiate into effector cells, extravasate, and subsequently infiltrate the transplant [7, 17]. Different chemokines, cytokines, and the upregulation of vascular adhesion molecules guide this process [18], finally leading to necrotic parenchymal destruction or initiation of apoptosis [19, 20].

Owing to modern efficacious immunosuppressants, the clinical presentation of renal allograft rejection episodes has changed from fever and graft tenderness to an often subclinical manifestation characterized by increasing proteinuria, elevation of serum creatinine, or solely histological changes, which hinders early recognition [21].

Gold standard for diagnosis of allograft rejection is the histopathologic evaluation of core needle biopsies by applying international consensus criteria, the Banff classification [21]. The biopsy procedure potentially causes serious complications for the allograft recipient, and it also underlies distinct contraindications. Therefore, noninvasive techniques to detect AR would be a key advancement for this field.

Apart from structural details, ultrasound- and MRI-based techniques nowadays are also capable to visualize functional and biological processes to some extent in the context of the abovementioned inflammatory processes that accompany AR.

As transplant recipients require active surveillance and frequent assessments of the graft's condition, we provide a review of the current nonradiation-based, noninvasive imaging techniques to detect AR that might help reduce the need for biopsies in the future. All technical approaches bare the potential to be used (in a modification) for the diagnoses of AR in other transplant organs than the kidney as well.

## 2. Ultrasound

Over the last four years, significant progress has been made particularly in the field of ultrasound- and MRI-based techniques. Sonographic examination is one of the most valuable clinical tools for patients' assessment. It is widely available and routinely utilized to monitor specific properties such as perfusion, resistance indices etc., of the graft after organ transplantation. The parameters assessed include unspecific features of AR such as enlargement of the transplant caused by swelling, abatement of corticomedullary differentiation, change in echogenicity, and distinctive structures such as medullary pyramids. Analysis of the perfusion of the grafts can be performed with Doppler ultrasound and contrast-enhanced ultrasound (CEUS) examination. Sonography is advantageous due to its innocuousness, cost-effectiveness, and wide availability. However, on the downside, it has limitations with regard to sensitivity and specificity for AR until now.

Recent approaches have addressed these limitations and offered potential solutions to overcome them. One of those approaches is the assessment of the resistive index (RI) [22], which increases when grafts undergo antibody-mediated rejection. While using the RI, one has to keep in mind that it is increased in cases of acute tubular necrosis (ATN) as well, and the systemic resistance of the vasculature, pulse pressure, and heart rate and rhythm also contribute to it. Thus, RI cannot be precisely assessed in patients with atrial fibrillation for instance, and it is significantly influenced by the recipient's age or dialysis time before the transplantation. While it is associated with overall survival, its usefulness for the prediction of graft survival is questionable [22–24].

**Figure 1:**
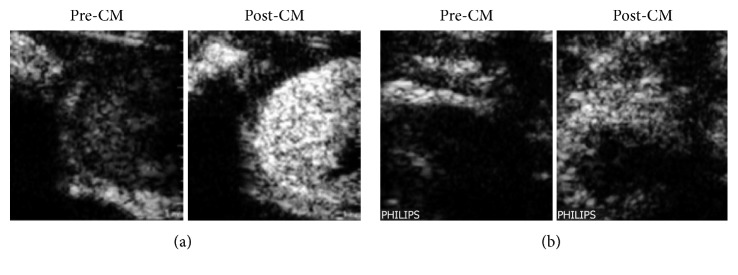
Representative ultrasound images of an allogeneic kidney transplantation in a rat (atx, allograft) (a): its native control kidney (native)(b) 4 days after transplantation before (pre-CM), 15 minutes after tail vein injection of microbubbles labeled with an antibody targeted against CD3 positive T cells (post-CM). CM: contrast media/microbubbles conjugated with anti-CD3 antibody [28].

A further ultrasound-based approach uses CEUS, which involves the use of either intravenously applied microbubble-based contrast agents to increase the echogenicity of blood or targeted microbubble-based contrast agents addressing specific tissues. CEUS parameters of interest are rising time, time to peak, and delta-time among other regions of interest [25]. Conventional CEUS cannot be used for the definitive detection of AR, since the increase in the echogenicity of blood is the only indicator. It is capable to visualize and quantify renal perfusion abnormalities suspicious of acute vascular rejection, but not sufficient to distinguish these abnormalities from other causes of abnormal perfusion [26]. A more specific detection of AR becomes a possibility when microbubble-based contrast agents targeted against T cells are used. By labeling the contrast agent with specific antibodies (for instance, against T cell surface antigen CD3 or other T cell surface antigens like CD8 or CD4), the echogenicity of the graft can be sufficiently increased to enable the reliable diagnosis of AR (Figure 1) [27].

The acquired ultrasound signal intensities also increase with the severity of inflammation, indicating a possible measure of the degree of AR with this method. Furthermore, through targeted CEUS, it was possible to differentiate AR from ATN and acute calcineurin inhibitor toxicity. CEUS demonstrated the capability of therapeutic monitoring of the immunosuppressive treatment of manifest AR, as signal intensity distinctly decreased 24 hours after beginning of rejection therapy.

Therefore, since the pathological mechanism of acute T cell mediated rejection can be observed in cases of AR in other transplanted solid organs, CEUS can potentially detect AR in other organs as well [27].

Liao et al. demonstrated in a rat study that C4d can also serve as a target for labeled microbubbles to detect and visualize C4d deposition by means of CEUS in glomeruli and peritubular capillaries as a characteristic of antibody-mediated rejection (AMR) [29], which represents the leading cause of kidney allograft loss [30]. This method also allows quantitative analysis of C4d deposition via normalized intensity differences (NID) [29].

However, for the translation of these approaches to the human body, the immunogenic streptavidin-based conjugation between the microbubble-based contrast agent and the appropriate antibody requires modification.

In another recent study, Meier et al. investigated if a newly developed serial duplex index (SDI) can be used to differentiate between AR and acute vascular rejection more effectively in comparison with the established Doppler parameters RI and pulsatility index (PI) in the first days after the transplantation [31]. The PI represents a Doppler derived index that depends on downstream renal artery resistance and stiffness [32]. Based on the parameters RI, PI, and cortex-pelvis proportion (CPP) calculated on the day of the biopsy (*t*_0_) and 3 to 7 days before the biopsy, the SDI was calculated as RI ratio × PI ratio/CPP ratio. A retrospective analysis of 121 patients revealed that the SDI was significantly different between patients with normal graft function, acute cellular rejection, and acute vascular rejection. The RI and PI ratios were significantly different only between patients with normal graft function and acute vascular rejection. The developed SDI was able to detect acute renal transplant rejection with greater sensitivity and specificity than the RI and PI ratios, thus it might be helpful to indicate renal biopsy in the future [31]. Nevertheless, this method lacks prospective evaluation.

A different ultrasound-based technique, evaluated to detect AR, was described by Jiménez et al. in 2016. They performed real-time contrast-enhanced sonography (RT-CES) to investigate cortical capillary blood flow (CCBF) after kidney transplantation. RT-CES provides an analysis and quantification of vascular refilling in any region of interest (for instance, the renal cortex) by depicting the destruction of injected microbubbles through an ultrasound pulse. The refilling of a certain area is an indicator of tissue perfusion. A secondary aim was to explore the influences of AR, acute tubular necrosis, and calcineurin inhibitor toxicity on CCBF, even though this study was not designed to specifically determine these differences [33].

Although AR is associated with lower CCBF in general, CCBF did not demonstrate sufficient sensitivity to distinguish rejection due to tissue edema and cellular infiltration [34]. Unfortunately, RI and CCBF did not correlate with any of the analyzed timepoints (48 h, 5–7 days, and 1, 3, and 12 months after transplantation), possibly because CCBF reflects both donor and graft characteristics, as it can be deduced by the CCBF correlation with donor's age, AR episodes, and living vs. brain-death donor. The authors hypothesized that CCBF would reflect the basal vasculature state from the donor early after transplantation, and later this state would be changed by inflammatory events after transplantation [33].

Yang et al. utilized point shear wave elastography (p-SWE) based on acoustic radiation force (ARF) impulse to quantify tissue stiffness by measuring shear wave speed (SWS) in a prospective study with 115 KTx recipients [35]. The technical principle was already established for detection and quantification of liver fibrosis [36]. The shear wave speed was found to be significantly higher in patients with AR than in the non-AR patients. The authors invented a model called the SEV index that comprised of the parameters SWS, estimated glomerular filtration rate, and kidney volume change for noninvasive detection of AR. In kidneys undergoing AR, SWS was significantly increased (reflecting an increase in parenchymal stiffness) as compared to non-AR kidneys, including stable functioning grafts and ATN kidneys. Thus, SWS analysis for the detection of AR might hold potential. Since kidney volume significantly increases in patients with AR, the authors hypothesized that the edema formed during AR could increase intrarenal pressure due to the strong fibrous capsule surrounding the kidney. This mechanism might be responsible for the observed increased stiffness. In contrast to AR, ATN, which is another common cause for delayed graft function, was additionally associated with a significantly decreased graft volume [35].

A more recent prospective study to evaluate the value of SWS for the differentiation of stable allograft function from acute and chronic allograft dysfunction was performed by Ghonge et al. The study revealed that SWS can help to differentiate stable allograft function from acute and chronic dysfunction in addition to laboratory and Doppler-based parameters [37].

Kim et al. investigated the worthwhile issue of diagnosing subclinical kidney allograft rejection (SCR) in stable functioning grafts by quantifying tissue elasticity with SWE to identify rejection episodes at a stage with better treatment options. They examined 95 patients who underwent protocol biopsies either 10 days or 1 year after Tx and exhibited a stable allograft function. 34 of them showed histological characteristics of acute rejection. The authors could demonstrate that tissue elasticity was significantly increased in patients with SCR compared to those without in univariate analysis, without being an independent predictor of SCR in multivariate analysis. No differences in tissue elasticity could be found between the histological subtypes of rejection [38]. Important but difficult to control counfounding factors of quantitative SWE measurement are amongst others different depths of the allograft and its movement, different pressures of the transducer, and the exact incident angle of the acoustic beam [38, 39].

Viscoelastic response (VisR) ultrasound is an alternative ARF-based tool using two co-localized ARF impulses to delineate tissue properties by measuring viscosity and elasticity (for details, see [40]). Hossain et al. performed a prospective study with 44 patients to examine the ability of VisR to evaluate renal transplant status. This study revealed the feasibility of VisR to significantly differentiate between control allografts and those harboring pathological features. Nevertheless, it was not sufficient to discriminate specifically between various graft pathologies. Hence, VisR could help to preserve patients without a structural graft pathology against unnecessary biopsies but is not able to replace biopsies in patients showing a suspicious VisR finding [41].

## 3. MRI

The natural magnetic properties of hydrogen nuclei can be detected in a magnetic field with the use of magnetic resonance imaging (MRI). The strength of MRI lies in its excellent intrinsic soft tissue contrast, unlimited penetration depth, and high anatomical resolution in addition to the functional assessment of the graft. The technique is capable of differentiating tissue characteristics based on intrinsic MR properties such as *T*_1_ and *T*_2_ relaxation times, water content, and diffusivity [42]. Thus, MRI facilitates the detection of distinctive features of vascular and interstitial structures.

Different MRI techniques have already been successfully applied to discriminate between different causes of renal allograft injury such as AR and ATN, and they allow (to some extent) the visualization of the pathophysiological processes underlying the respective type of injury [42, 43].

### 3.1. Renal Function

A common MRI method to assess renal function is dynamic contrast enhanced MRI (DCE MRI). It depends on gadolinium-based contrast agent protocols, and it is also termed MR renography (MRR). The contrast agents used in this technique are freely filtered across the glomeruli yet not secreted or reabsorbed in the tubules ideally. Due to these specific characteristics, it optimally assesses renal perfusion, glomerular filtration rate (GFR), and tubular function that has been proved useful for discrimination between AR and ATN [43]. One pathological feature of renal grafts undergoing AR is a significantly reduced cortical and medullary blood flow compared to nonrejected grafts [44–47]. The reduced medullary blood flow in grafts with AR seems to be characteristic to distinguish between AR and ATN in particular [48].

Yamamoto et al. proved the feasibility of discrimination between several types of allograft impairment using a new quantitative analysis method of MRR. They implemented a multicompartmental kinetic kidney model to determine the mean transit time (MTT) of a contrast agent through the different compartments of the kidney. Even though some significant differences in the fractional MTT values between normal grafts or grafts undergoing AR or ATN were obtained, substantial overlaps were observed when these groups were compared with themselves and with healthy control kidneys [47].

Notably, the application of gadolinium-based MRI to patients with severely impaired renal function potentially accompanies the rare but deleterious side effect of contrast-induced nephrogenic systemic fibrosis [49]. Moreover, recent findings revealed the association of intravenous exposure to especially linear gadolinium-containing complexes with neuronal tissue deposition in patients with normal renal function as well. The clinical significance of that finding is undiscovered so far [50].

**Figure 2:**
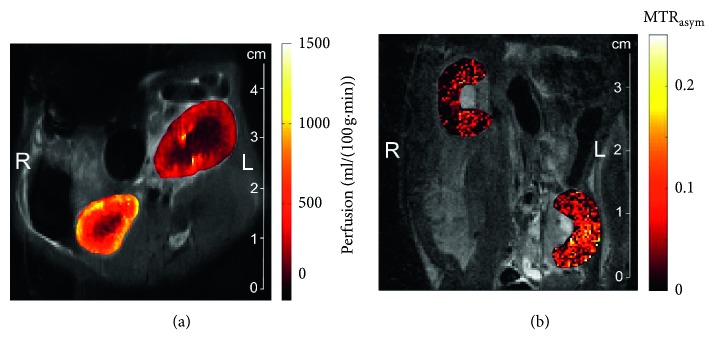
Representative in vivo ASL perfusion (a) and glucoCEST MTR_asym_ (b) maps of the renal cortex and medulla of an allogeneic transplantation in a rat on day 4 posttransplantation, showing the renal allograft undergoing an acute cellular rejection on the right side (L) and the healthy right contralateral kidney on the left side (R).

In contrast, arterial spin labeling (ASL) is an MRI tool that utilizes arterial blood flow as an endogenous contrast agent, and it allows the study of allograft function, particularly by longitudinal perfusion evaluation. It comprises of two acquisitions, one labeled by modifying the longitudinal magnetization of arterial blood water and a control acquisition obtained without arterial labeling. The labeled protons find their way via the arterial bloodstream to the targeted tissue, where they pass from the intra- to the extravascular compartment and reduce the equilibrium magnetization slightly by a few percent. Imaging is performed at time *T*_I_ (inversion time) after the pulsed labeling with the use of a rapid imaging technique, owing to the time required for the labeled protons to perfuse the tissue. In the control acquisition, arterial protons at the target structure are relaxed and in equilibrium. Methods for ASL and labeling pulses can be categorized into continuous (CASL), pulsed (PASL), and velocity-selective (VSASL) techniques [51]. The subtraction of the labeled and control acquisitions suppresses the signal from the static tissue and provides a perfusion-weighted image. Quantitative perfusion maps can be calculated with the use of various TIs (Figure 2(a)). ASL studies using a flow-sensitive alternating inversion recovery (FAIR-ASL) scheme (for details about FAIR-ASL, see [52]) reveal a significant lower perfusion in allografts vs. native kidneys [53]. In conjunction with this, renal allografts with acute decrease in renal function showed a significant lower cortical perfusion when compared to those with steady function in the long-term and the postoperative period [54]. However, the underlying disease leading to the decrease in perfusion has not been identified.

Another approach for the functional examination of transplanted kidneys to distinguish between AR and ATN is exploitation of the paramagnetic properties of deoxyhemoglobin via blood-oxygen level dependent (BOLD) MR [55–58]. Deoxyhemoglobin accumulates in tissues with lower oxygen concentration. It is strongly paramagnetic due to its unpaired electrons at the iron centre and thus leads to a shortened transverse relaxation time constant *T*_2_^*∗*^. Inversely, the apparent relaxation rate *R*_2_^*∗*^(=1/*T*_2_^*∗*^) is elevated. In this context, BOLD MR is feasible in providing information about renal parenchymal oxygen concentration [55]. In AR kidney allografts, medullary *R*_2_^*∗*^ values decrease significantly, corresponding to a higher oxygenation in comparison with kidneys with ATN. Interestingly, this observation was accompanied by a reduced medullary blood flow assessed by perfusion MR which appears contradictory [55]. The increase in oxygen consumption by active tubular reabsorption whenever filtration and blood flow rise together may be a causal factor for this. As a consequence, regional oxygen tension is not as strongly associated with regional blood flow as in other organs [59].

### 3.2. Renal Structure and Morphology

Diffusion-weighted MRI (DWI MRI) is a contrast agent-independent MRI technique that depends on the signal decay induced by relative diffusion-based displacement of water molecules. It can be quantified by calculating the apparent diffusion coefficient (ADC). The ADC is influenced by the tissue microstructure and separated from the directionality of molecular motion, thus ADC values represent a measure for tissue diffusivity [60, 61].

Hueper et al. recently performed a study wherein they investigated mice after isogenic and allogeneic KTx at day 1 and day 6 after the transplantation. They used a combination of both functional MRI techniques—DWI and mapping of *T*_2_-relaxation time (*T*_2_-mapping)—to investigate the severity and course of inflammation and edema formation following KTx during the development of AR and IRI.

The authors observed a progressive ADC reduction in allogeneic grafts when compared to isogeneic grafts and normal kidneys, which correlated to histologic findings of tissue inflammation corresponding to AR.

*T*_2_ relaxation times increased as a correlate for tissue edema, and this was observed in both transplantation groups. The authors assumed that the acute kidney injury of the graft following prolonged cold ischemia time was responsible for this finding. Interestingly, only the allogeneic group showed an abrogated *T*_2_-difference between the renal compartments, which indicates a disturbance of physiological differences of tissue water content.

Therefore, Hueper et al., who used a 7 T MRI scanner for their study, concluded that morphological MRI clearly allowed differentiation between allogeneic kidney grafts with AR and isogenic kidney grafts with IRI and provided detailed tissue information with regard to the graft using DWI and *T*_2_-mapping. In this case, one limitation might be that edema formation interferes with diffusion and T cell invasion which is able to alter *T*_2_-relaxation.

In order to translate this into clinical practice, two issues have to be considered: first, renal anatomy and physiology of mice differ from humans, and second, clinical MRI scanners usually remain in the range of 1.5–3T in contrast to 3–9.4T frequently used in rodent studies. Thus, MRI parameters may differ [62].

For imaging of renal structures, diffusion tensor imaging (DTI) is a more sensitive MRI-based approach that has been applied by Lanzman et al. By sampling several different diffusion directions, DTI addresses the issue of anisotropic diffusion properties due to the radial orientation of main anatomic structures like vessels and tubules. DTI is an effective tool for the assessment of the fractional anisotropy, which is a measure for directionality of diffusion in tissues [63, 64].

### 3.3. Molecular Imaging

Several studies have utilized nanoparticles to specifically detect immune cells or immune proteins in the kidney by using MRI to image the pathophysiological processes that occur when undergoing rejection (for a review, see [65]).

Hauger et al. and Chae et al. proposed application of superparamagnetic iron oxide (SPIO) particle-loaded macrophages to investigate native and transplanted kidney grafts. The first study mentioned was performed in human patients, and the latter in a rat model. Imaging of macrophage infiltration was performed three and five days after the application of the contrast agent. SPIOs cause local field distortion and result in a strong reduction of the relaxation time constant *T*_2_^*∗*^. Hauger et al. found a specific MR pattern for ATN that may be helpful in distinguishing ATN from AR. Furthermore, they were able to successfully discriminate between inflammatory and noninflammatory causes of kidney failure. However, the significant delay between contrast agent application and data acquisition is a strong limitation of this procedure. Moreover, imaging of phagocyte activity is unspecific, as these cells participate in different inflammatory events in the kidney. Chae et al. demonstrated macrophage homing using MRI in their allograft rejection model. However, other causes of graft failure besides AR were not investigated in their study. Unfortunately, nonphagocytic cells, such as T cells, which are more specific to AR, generally present a low SPIO-labeling efficiency and poor contrast agent uptake. Currently, this is a significant limitation to cellular MR imaging *in vivo* [66, 67].

Polyethylene glycol-coated superparamagnetic nanosized iron-oxide particles constitute a new synthesized class of MRI contrast agent. These particles were tested for the labeling of T cells in a rat model of AR after heart-lung transplantation with a T cell purity of about 90% [68]. This technique offers an approach to track nonphagocytic cells, such as T- and B-lymphocytes, and it may also potentially be translated into clinical application for detection of AR after KTx. It must be mentioned critically that the authors of this study did not regard the 10% non-T cells and their impact. In addition, the labeling is not permanent, and most of the labeling signal shown remains extracellular due to the sticky particles adhering to the plasma membrane of these cells.

Recently, we published a new MRI-based approach called glucoCEST for noninvasive and differential *in vivo* studies of renal allograft injuries. This technique aims to assess regional tissue glucose content. GlucoCEST uses D-glucose as a naturally occurring biodegradable MRI contrast agent, which can be monitored by chemical exchange saturation transfer (CEST) [69].

The measurement of AR-related regional glucose accumulation helped to differentiate AR from syngeneic grafts without AR, kidneys with IRI, and kidneys with cyclosporine A- (CsA-) induced toxicity. The technique involved the calculation of a MTR_asym_ contrast ratio of cortex to medulla, which was found to be significantly increased in AR compared to the other subgroups (Figure 2(b)). The differentiation of AR from major clinical differential diagnoses of delayed graft function like IRI and CsA toxicity is an important advancement of this new method. Additionally, this approach successfully monitored a response of AR to immunosuppressive treatment at an early point of time [70].

The feasibility of clinical translation for the glucoCEST technique has already been demonstrated in patients with glioma [71]. Therefore, we are convinced this could be a promising approach for noninvasive detection of AR in humans.

## 4. Conclusion

New innocuous and sensitive diagnostic tools for the detection of AR assessing the whole organ, particularly in differential diagnostics of delayed graft function, are highly desired. Two particularly suited applications to address this issue are ultrasound and MRI. Advances in technology and contrast agent development have opened new possibilities in this regard. At present, all of these promising new technologies are still at an experimental stage and have yet either to be transferred from animal models to clinics or to be refined to prove an advantage over standardly applied core needle biopsy. For the moment, they are partly capable to better identify patients in need of a kidney allograft biopsy and they offer the potential to advance the clinical routine for noninvasive and specific diagnosis of AR as well as the longitudinal surveillance monitoring of the allograft. The biggest advantage of ultrasound-based diagnostics over MRI-based ones is their broad availability, their potential for bedside and real-time diagnostics, and the relatively low costs. However, in contrast to MRI, ultrasound-based diagnostics has a substantial interobserver variability and needs experienced investigators. Furthermore, most MRI sequences are already available for clinical scanners.

However, detection approaches for structural, morphological, or functional features by ultrasound (perfusion, arterial blood flow, stiffness, elasticity, and viscosity) as well as MRI (perfusion, arterial blood flow, tubular function, tissue diffusivity, and oxygen concentration) lack specificity for the detection of AR; imaging of characteristic molecular biological processes with both ultrasound (T cell migration and C4d deposition) and MRI (macrophage infiltration, T cell migration, and glucose accumulation) bares the potential to overcome this hurdle.

Of course, rejections can occur in every allogenic transplant. Accordingly, the reviewed techniques for noninvasive detection of renal allograft rejection are principally adaptable to other allogenic transplanted solid organs.

## Abbreviations

ADC: Apparent diffusion coefficient
ARF: Acoustic radiation force
ARFI: Acoustic radiation force impulse imaging
AMR: Acute antibody-mediated rejection
AR: Acute rejection
ASL: Arterial spin labeling
ATN: Acute tubular necrosis
BOLD: Blood-oxygen level dependent
CCBF: Cortical capillary blood flow
CD: Cluster of differentiation
CEST: Chemical exchange saturation transfer
CEUS: Contrast-enhanced ultrasound
CPP: Cortex-pelvis proportion
CSA: Cyclosporine A
CT: Computed tomography
CTL: Cytotoxic T-lymphocytes
DCE: Dynamic contrast enhanced
DTI: Diffusion tensor imaging
DWI: Diffusion-weighted
ESRD: End-stage renal disease
FAIR-ASL: Flow-sensitivity alternating inversion recovery
GFR: Glomerular filtration rate
HLA: Human leukocyte antigen
IRI: Ischemia-reperfusion injury
KTx: Kidney transplantation
MR: Magnetic resonance
MRI: Magnetic resonance imaging
MRR: Magnetic resonance renography
MTT: Mean transit time
NID: Normalized intensity differences
PI: Pulsatility index
p-SWE: Point-shear wave elastography
RI: Resistive index
RT-CES: Real-time contrast-enhanced sonography
SCR: Subclinical kidney allograft rejection
SDI: Serial duplex index
SPIO: Ultrasmall supermagnetic iron oxide
SWEI: Shear wave sonoelastography
SWS: Shear wave speed
T: Tesla
TCMR: T cell mediated rejection
TI: Inversion time
VISR: Viscoelastic response ultrasound.
